# Supplementary material for: Femtosecond structural transformation of phase-change materials far from equilibrium monitored by coherent phonons
Source: Nat Commun. 2015 Sep 25;6:8367. doi: 10.1038/ncomms9367 (PMC4598557; doi:10.1038/ncomms9367)
Supplement: Supplementary Information — Supplementary Figures 1-6, Supplementary Notes 1-3 and Supplementary References [file ncomms9367-s1.pdf]

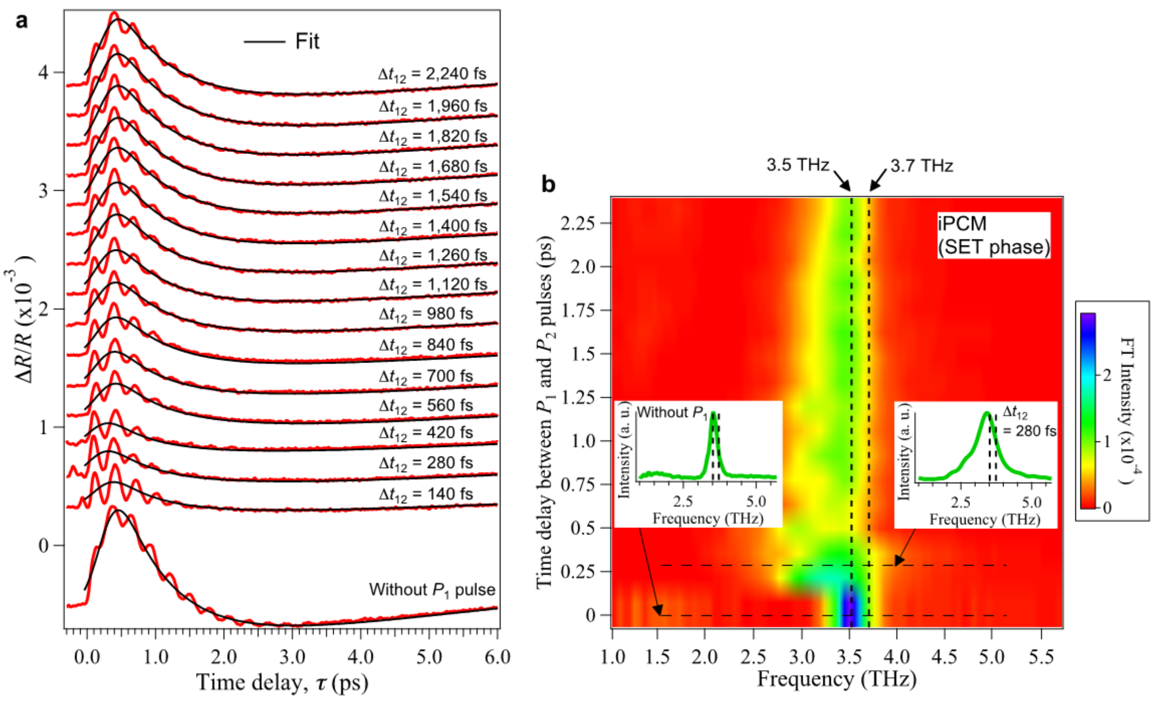

Supplementary Figure 1. Transient reflectivity obtained by a homodyne detection technique. (**a**) Raw transient reflectivity changes observed in the SET phase of iPCM for different separation times Δt_{12} at the constant pump fluences of P_1 = 10.6 mJ cm^{-2} and P_2 = 2.0 mJ cm^{-2}, by using a homodyne detection technique (by modulating the P_2 pump pulse with an optical chopper at 2 kHz), as described in Ref. [1]. The trace at the bottom represents the data for single pulse excitation with only P_2 without P_1 pulse. (**b**) The corresponding two-dimensional plot of FT spectra obtained from the time-domain data in (**a**) after subtraction of the slowly varying carrier background, which is presented by the fit line in (**a**). The vertical dotted lines represent the peak positions before the irradiation of P_1 pulse (3.5 THz) and the peak position of the blue shifting (3.7 THz) observed only in the heterodyne detection presented in Figs. 2 and 3 in the main text. The FT spectra in the inset correspond to the slices for the case without P_1 pulse (left side inset) and the case of Δt_{12} = 280 fs (right side inset), respectively. The homodyne detection data with much lower P_2 pump fluence demonstrate that the frequency blue-shift (phonon hardening) was not observed.

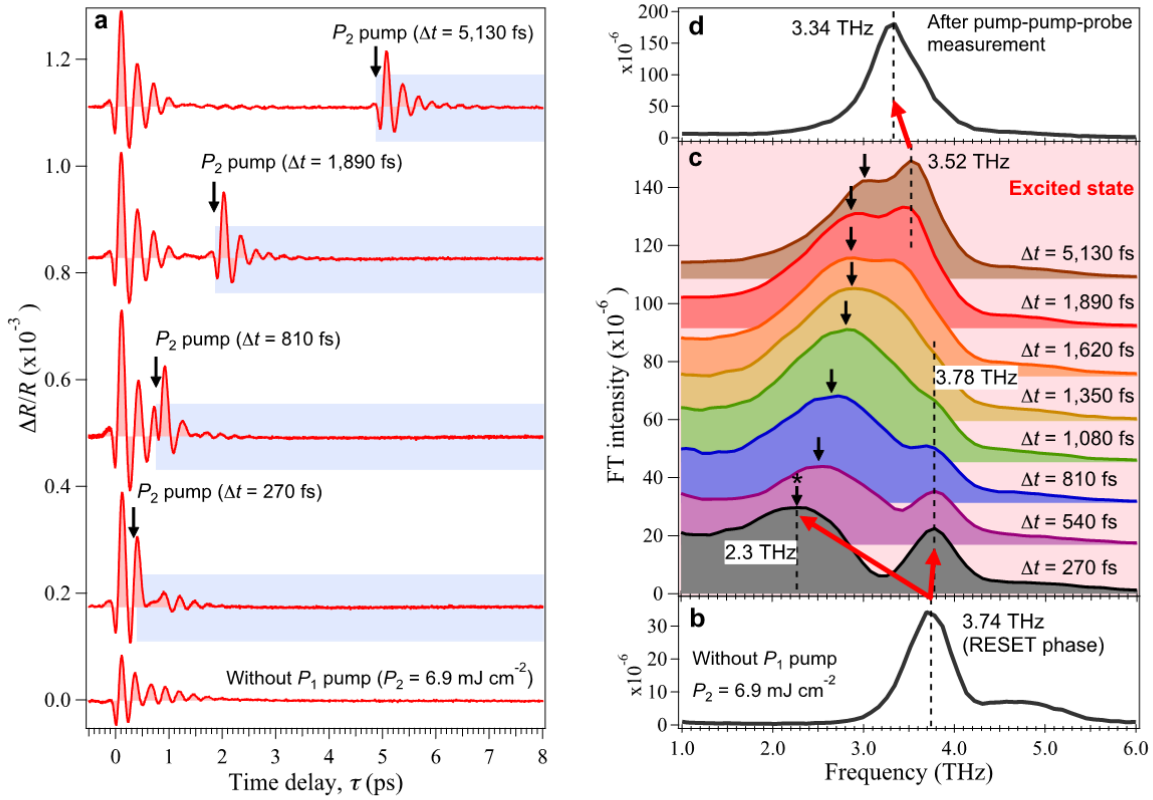

Supplementary Figure 2. Time-domain coherent phonon responses and corresponding FT spectra for the RESET phase of iPCM. (**a**) Transient reflectivity trace observed in the RESET phase of iPCM film at Δt = 270 fs, 810 fs, 1,890 fs, and 5,130 fs with P_1 = 10.6 mJ cm^{-2} and P_2 = 6.9 mJ cm^{-2}. The light-blue rectangles represent the coherent phonon signal used for monitoring the excited lattice and converted into FT spectra. The result for the case without prepump (P_1) is shown at the bottom for reference. (**b**) FT spectrum in the RESET phase monitored by the same conditions in Fig. 3a. The dotted line in (**b**) corresponds to the frequency of the optical mode in the RESET phase (Ω_{RESET} = 3.74 THz). (**c**) FT spectra obtained from the time-domain data in the excited state at various Δt. The red arrows show the split of the optical mode into two peaks at 3.78 THz and 2.3 THz at Δt = 270 fs. The dotted lines in (**c**) correspond to a dynamic shift of the 3.74 THz peak via 3.78 THz down to 3.52 THz at Δt = 1,890 fs. The vertical black arrows point out the peak shift of the 2.3 THz mode. (**d**) FT spectrum observed after the measurement of Δt = 5,130 fs in (**c**) monitored by the same condition in (**b**). The dotted line in (**d**) is located at Ω_{new} = 3.34 THz, indicating the system transferred to a new metastable structure, whose frequency is similar to the case of the SET phase, and irreversible phase change in the RESET phase of iPCM is demonstrated.

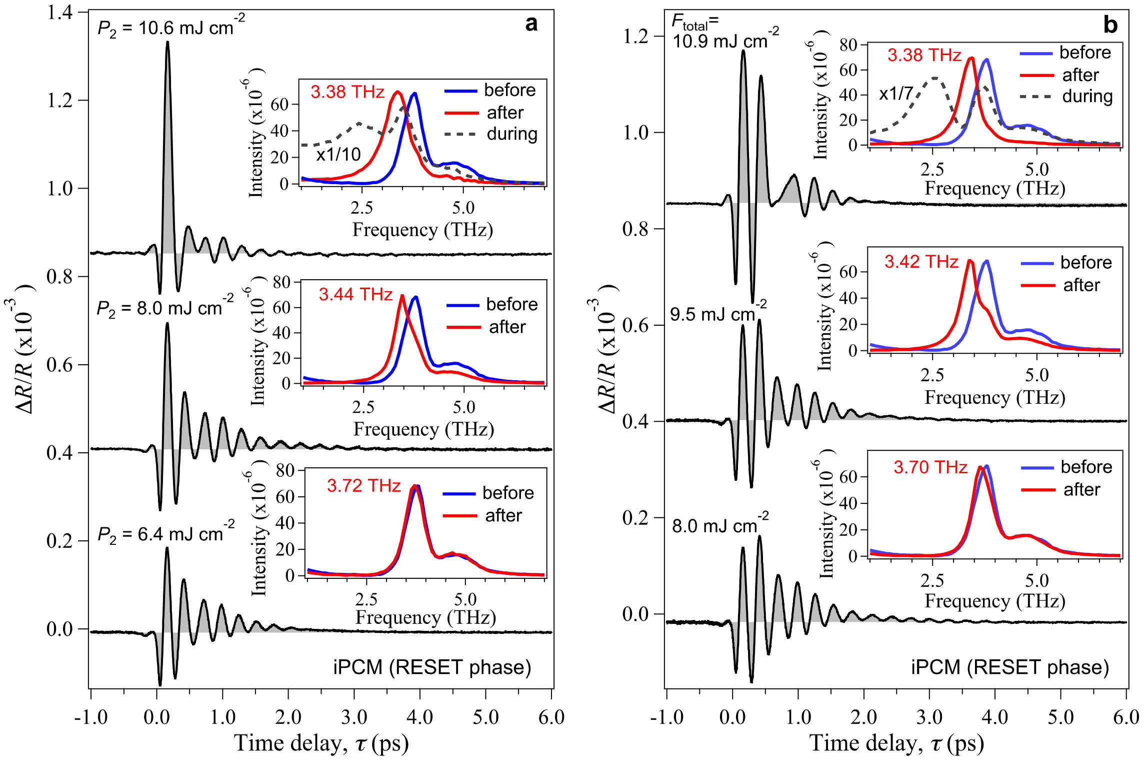

Supplementary Figure 3. Investigation of threshold for the irreversible phase change in the RESET phase of iPCM. (**a**) The transient reflectivity changes observed in the RESET phase of iPCM at room temperature. The pump fluence was varied in the vicinity of the threshold for irreversible phase change from 6.4 to 10.6 mJ cm^{-2} under the single pulse excitation with only P_2 pulse. The insets represent the FT spectra obtained before and after irradiation by the different fluences, recorded using the single P_2 pulse at 3.2 mJ cm^{-2}. The FT spectrum presented by the dashed line on the top inset was obtained from a reflectivity signal with P_2 = 10.6 mJ cm^{-2}, meaning it reflects the state during the irreversible phase change. The peak frequency presented by red text is obtained by the FT spectra after irradiation by the P_2 pulse. The peak position of the optical mode, involving covalently bonded Ge atoms, is found to be irreversible when $P_2 \geq 8.0$ mJ cm^{-2}. (**b**) The same as (**a**), but obtained by using irradiation by a two-pulse sequence with Δt = 270 fs for a total pump fluence ranging from 8.0 to 10.9 mJ cm^{-2} (a constant ratio of P_1:P_2 = 10:6.5 was maintained). The FT spectrum presented by the dashed line was obtained from the reflectivity signal just after the second pump pulse with F_{total} = 10.9 mJ cm^{-2}, meaning it reflects the state during irreversible phase change. The peak frequency presented by red text is obtained by the FT spectra after the irradiation by the double pump-pulse sequence. The peak position of the optical mode was found to be irreversible when $F_{total} \geq 9.5$ mJ cm^{-2}. Note that the FT spectra "during" the irreversible phase change exhibit different peak positions and intensities as shown in (**a**) and (**b**), implying that the pre-phase transformation state from the RESET phase is different for the case of a single pulse and a double pump-pulse sequence.

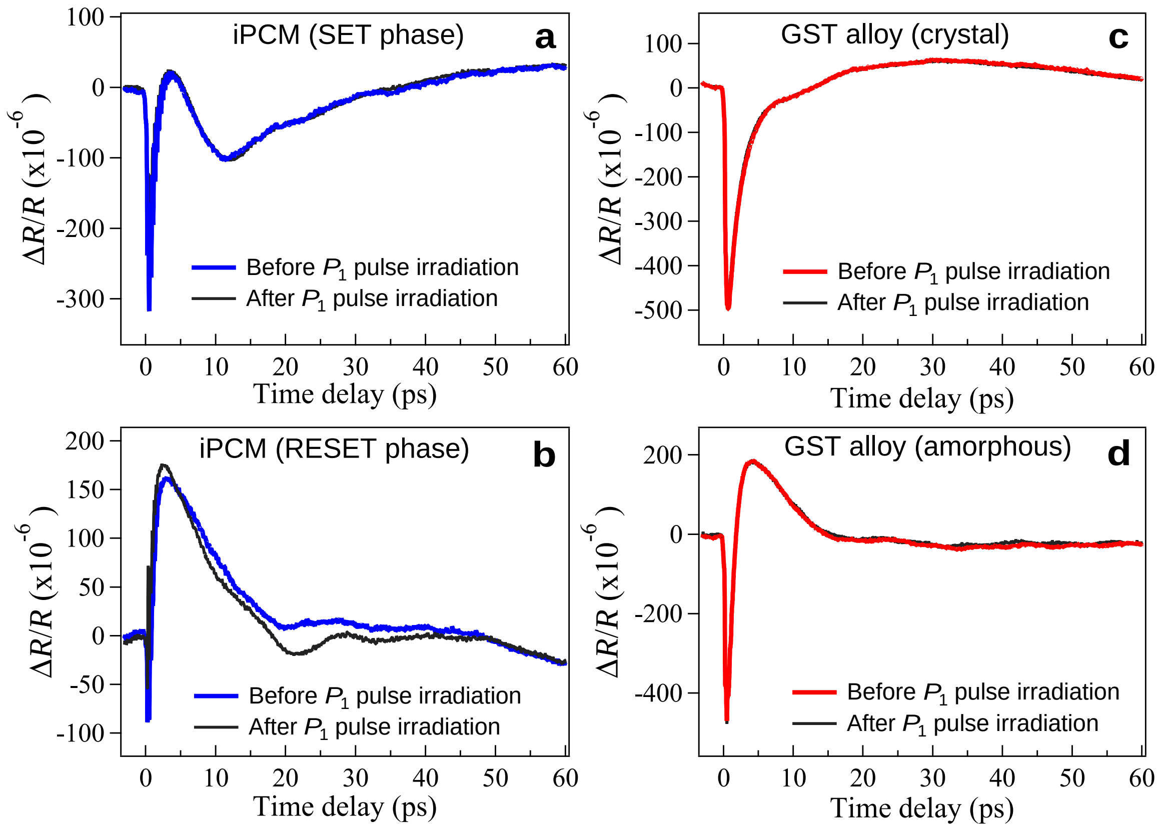

Supplementary Figure 4. Comparison of reflectivity change (carrier) responses for the SET and RESET phases of iPCM and GST alloy films obtained before and after irradiation by a P_1 pulse. P_1 = 10.6 mJ cm^{-2} and P_2 = 6.9 mJ cm^{-2} was maintained for all samples of the (**a**) SET phase of iPCM, (**b**) RESET phase of iPCM, (**c**) poly-crystalline (fcc) GST alloy, and (**d**) amorphous GST alloy. In the RESET phase of the iPCM, (**b**), there was a difference between the reflectivity response before and after irradiation by the P_1 pulse.

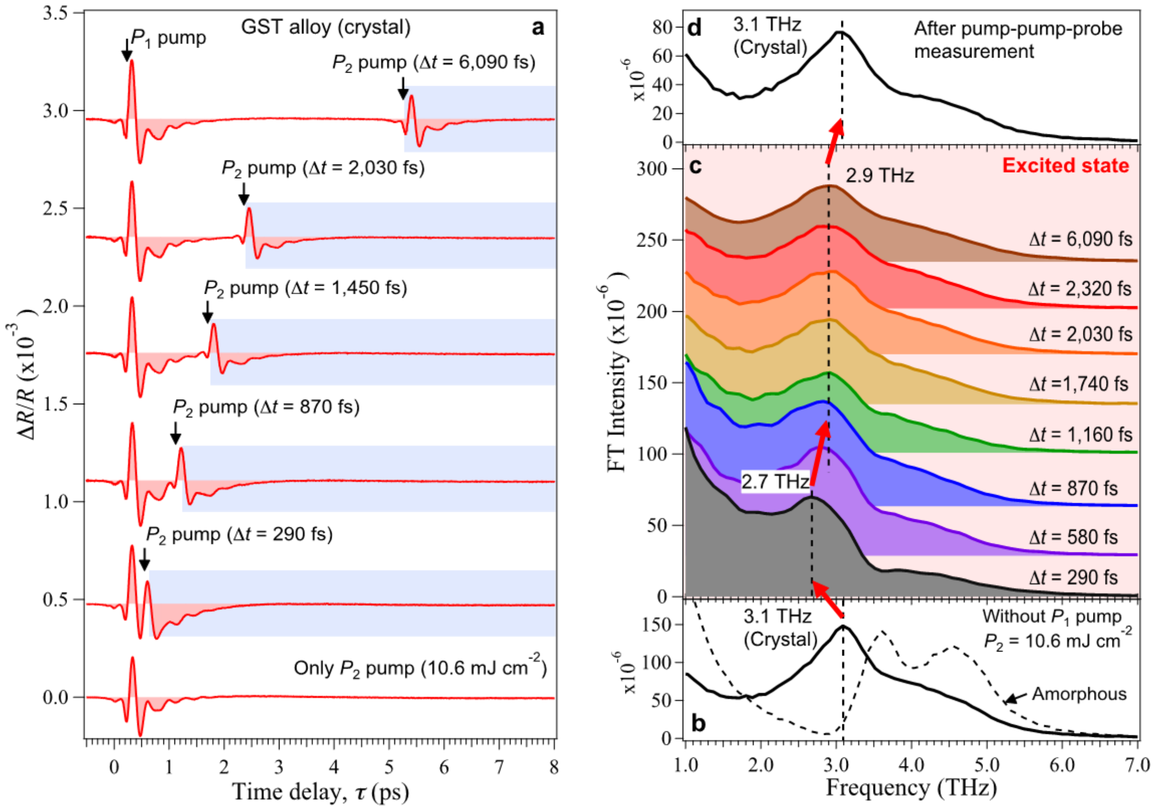

Supplementary Figure 5. Time-domain coherent phonon responses and corresponding FT spectra for the SET phase of GST alloy. (**a**) Transient reflectivity change observed in the poly-crystalline GST alloy film (30 nm thick) with slightly higher fluences of P_1 (16 mJ cm^{-2}) and P_2 (10.6 mJ cm^{-2}) pulses. The value of Δt was chosen to have the same separation time as used in iPCM experiment, since the original frequency at the lowest fluence limit was 3.3 THz (≈ 300 fs) for the poly-crystalline GST alloy. (**b**) FT spectrum observed without prepump (P_1) in a poly-crystalline GST alloy film (30 nm thick) at P_2 = 10.6 mJ cm^{-2}. The main peak position appears at 3.1 THz. The FT spectra observed in the amorphous phase of GST alloy film is also shown by the dashed line for reference. (**c**) FT spectra obtained from the time-domain data in the excited state at various Δt. The red arrows show the dynamic shift of the optical mode from 3.1 THz to 2.7 THz at Δt = 290 fs, followed by recovery to 2.9 THz at Δt = 870 fs. (**d**) FT spectrum observed without P_1 excitation at the same spot after the measurement for Δt = 6,090 fs in (**c**). The dotted line in (**d**) is located at 3.1 THz, indicating the system reverts to the initial crystalline phase.

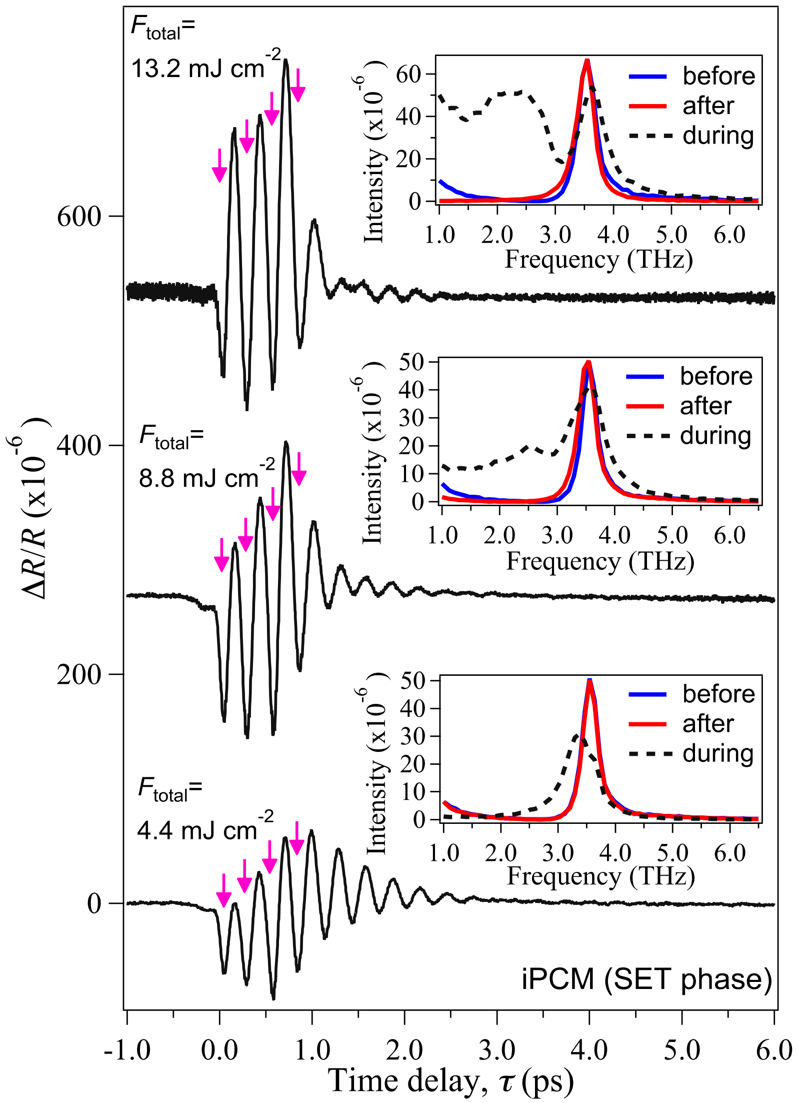

Supplementary Figure 6. Attempt of coherent control of non-thermal pre-phase transformation based on quadruple pulse excitation in the SET phase of iPCM film. The reflectivity data were taken at different excitation levels using a quadruple pulse sequence with a separation time of Δt = 290 fs at a repetition rate of 30 kHz. The four repetitive arrows indicate the time delay, at which the pump-pulses arrived. The insets represent the FT spectra obtained by using only P_2 = 2.6 mJ cm^{-2} pulse before and after irradiation by the quadruple pulse excitation. The frequency of the optical mode was not changed before and after the quadruple pulse excitation, staying at 3.55 THz. The FT spectra presented by dashed lines were obtained from the reflectivity signal during the quadruple pulse excitation. They exhibit emergence of the double peak spectra and blue-shift of the optical mode at F_{total} = 13.2 mJ cm^{-2} (P_1 = 4.0 mJ cm^{-2}, P_2 = 2.6 mJ cm^{-2}, P_3 = 4.0 mJ cm^{-2}, and P_4 = 2.6 mJ cm^{-2}) but the final state is still the same as that before the irradiation, meaning a reversible pre-phase transformation took place.

Supplementary Note 1.

Transient reflectivity studies of the RESET phase of iPCM. In contrast to the reversible dynamics observed in the SET phase, the phase transformation from the RESET phase of the iPCM was irreversible, as reported in Supplementary Fig. 2. Given the fact that this is an irreversible process, the sample has most probably been transformed following the first pair of pulses. Therefore, the initial state may be actually characterized by the top spectra in Supplementary Fig. 2d if one could observe the phonon spectra with a single shot, and not the bottom one in Supplementary Fig. 2b. From the spectra and systematics one could speculate that this state is actually a SET state, however according to Supplementary Fig. 4 and the related discussion this is not the case. If the phase transformation by the first pair of pulses was indeed the case, this would mean that multiple pump-pulse sequences could be used to switch iPCM from a new metastable structure, whose frequency is similar to the case of the SET phase (Supplementary Fig. 2d), after the first pair of pump-pulses, to the transient double-peak structure (Supplementary Fig. 2c) and back again to the metastable state similar to the SET phase (Supplementary Fig. 2d), within a single cycle of the pump-pump-probe sequence.

Fluence dependence in the RESET phase of iPCM. Supplementary Fig. 3 demonstrates the experimental results to check if only single pulse excitation can induce the irreversible phase change in the RESET phase of iPCM. It was found that a single pulse with a fluence higher than 8.0 mJ cm^{-2} could switch the RESET phase to a metastable state similar to the SET phase (Supplementary Fig. 3a). On the other hand, for the double pump-pulse sequence with a total fluence of $\geq$ 9.5 mJ cm^{-2} one can also switch the RESET phase to the same final state (Supplementary Fig. 3b). Thus, both the single and two-pump sequence can switch the RESET phase with similar total fluences.

The technique presented here can only be used to study reversible processes, and therefore to explore the precise pathway of the irreversible phenomena from the RESET phase of iPCM, a single-shot experiment taken for example by a technique such as X-ray diffraction or X-ray absorption will be required in the future.

Supplementary Note 2.

Reflectivity carrier response in different phases. To explore if the structural change observed is reversible, we have measured the reflectivity response for the amorphous and crystalline phases of a GST alloy film, and the SET and RESET phases of a iPCM film,

before and after irradiation by a strong P_1 pulse for Δt = 290 fs (for the SET phases) or 270 fs (for the RESET phases) as shown in Supplementary Fig. 4. The two different phases of the iPCM and GST alloy films exhibited very different carrier response. For the case of the response from the RESET phase of iPCM after irradiation by the P_1 pulse (Supplementary Fig. 4b), a significantly different response from that obtained before the irradiation was observed, while in other cases the responses exhibit a reversible nature as demonstrated in Supplementary Figs. 4a, 4c, and 4d. The small but significant deviation of the response in the RESET phase of the iPCM from that before the P_1 pulse irradiation implies that the superlattice structure may be preserved with small local atomic rearrangements during irradiation, although a more independent analysis of that state is required. Thus, the carrier responses shown in Supplementary Fig. 4 suggest the metastable state after irradiation by the P_1 pulse to the RESET phase of iPCM is different from that of the so-called laser-crystallized (LC) structure[2], which should show much different carrier response from the original phase.

Supplementary Note 3.

Transient reflectivity studies of the poly-crystalline (SET phase) GST alloy. To compare the $\Delta R/R$ signal observed in iPCM with that occurring in conventional GST alloy films, Supplementary Fig. 5a presents the transient reflectivity detected in a thin film (30 nm) of the poly-crystalline (fcc) phase of the GST alloy after irradiation with different Δt with slightly higher pump fluences. The coherent phonon oscillation exhibited a shorter lifetime even without prepump-pulse (P_1) excitation (see the bottom curve), and a further strong damping of the phonon oscillation was observed when the P_1 pulse was applied for different Δt. For the case of the poly-crystalline (SET phase) GST alloy, phonon frequency blue-shifting was not observed in the excited state, but broadening and red-shift (phonon softening) of the main peak (3.1 THz in Supplementary Fig. 5b) down to 2.7 THz was observed at Δt = 290 fs (Supplementary Fig. 5c). This phonon softening relaxes within a few picoseconds, followed by the full recovery of the original crystalline phase (Supplementary Fig. 5d). The broadening of the 3.1 THz peak in the excited state suggests transient disordering of the crystal lattice[3] and interactions with phonon modes associated with lattice defects or grain boundaries[4] leading to strong anharmonicity in the SET phase of the GST alloy[5], which results in faster phonon damping. From the fact that in the poly-crystalline GST alloy no frequency blue-shifting of the optical phonon was

observed, one cannot access the transient mixing state under the present double-pulse excitation for the case of the GST alloy film.

Supplementary References

[1] Yusupov. R. *et al.* Coherent dynamics of macroscopic electronic order through a symmetry breaking transition. *Nat. Phys*. **6**, 681–684 (2010).

[2] Hernandez-Rueda, J. *et al*. Coherent optical phonons in different phases of $Ge_2Sb_2Te_5$ upon strong laser excitation. *Appl. Phys. Lett*. **98**, 251906 (2011).

[3] Li, Xian-Bin. *et al*. Role of electronic excitation in the amorphization of Ge-Sb-Te alloys. *Phys. Rev. Lett.* **107**, 015501 (2011).

[4] Hase, M. *et al*. Dephasing of coherent phonons by lattice defects in bismuth films. *Appl. Phys. Lett.* **76**, 1258-1260 (2000).

[5] Matsunaga, T. *et al*. Phase-change materials: Vibrational softening upon crystallization and its impact on thermal properties. *Adv. Funct. Mater*. **21**, 2232–2239 (2011).
